# Risk Factors for Recurrence of Benign Paroxysmal Positional Vertigo. A Clinical Review

**DOI:** 10.3390/jcm10194372

**Published:** 2021-09-24

**Authors:** Ioanna Sfakianaki, Paris Binos, Petros Karkos, Grigorios G. Dimas, George Psillas

**Affiliations:** 1 1st Academic ENT Department, AHEPA Hospital, Aristotle University of Thessaloniki, 1, Stilponos Kyriakidi St., 546 36 Thessaloniki, Greece; g_sfakianaki@yahoo.com (I.S.); pkarkos@aol.com (P.K.); 2 Department of Rehabilitation Sciences, Cyprus University of Technology, Limassol 3036, Cyprus; paris.binos@cut.ac.cy; 3 1st Propaedeutic Department of Internal Medicine, AHEPA Hospital, Aristotle University of Thessaloniki, 1, Stilponos Kyriakidi St., 546 36 Thessaloniki, Greece; gregorydimas@yahoo.gr

**Keywords:** BPPV, BPPV recurrence, risk factors

## Abstract

Benign paroxysmal positional vertigo (BPPV) is one of the most common peripheral vestibular dysfunctions encountered in clinical practice. Although the treatment of BPPV is relatively successful, many patients develop recurrence after treatment. Our purpose is to evaluate the mean recurrence rate and risk factors of BPPV after treatment. A review of the literature on the risk factors of BPPV recurrence was performed. A thorough search was conducted using electronic databases, namely Pubmed, CINAHL, Academic Search Complete and Scopus for studies published from 2000 to 2020. Thirty studies were included in this review with 13,358 participants. The recurrence rate of BPPV ranged from 13.7% to 48% for studies with follow-up <1 year, and from 13.3% to 65% for studies with follow-up ≥2 years. Pathophysiologic mechanisms and implication of each of the following risk factors in the recurrence of BPPV were described: advanced age, female gender, Meniere’s disease, trauma, osteopenia or osteoporosis, vitamin D deficiency, diabetes mellitus, hypertension, hyperlipidemia, cardiovascular disease, migraine, bilateral/multicanal BPPV, cervical osteoarthrosis and sleep disorders. Patients with hyperlipidemia and hypertension had the highest recurrence rates of BPPV, 67.80% and 55.89%, respectively, indicating that vascular comorbidities increase the risk of BPPV recurrence. In addition, more than half of patients (53.48%) with diabetes mellitus and BPPV experienced recurrence of BPPV. Knowledge and awareness of risk factors for recurrence of BPPV are essential for the assessment and long-term prognosis of patients. Identification of these relapse risk factors may enhance the ability of clinicians to accurately counsel patients regarding BPPV and associated comorbidities.

## 1. Introduction

Benign paroxysmal positional vertigo (BPPV) is the commonest peripheral vestibular condition encountered in a neurotology clinic and it accounts for about 20% to 30% of all the vestibular complaints [1,2,3]. The mechanism of BPPV has been based on dislodged otoliths that leave the utricle and freely float in the semicircular canals or attach to the cupula, making the labyrinth sensitive to gravitational forces. BPPV is characterized by recurrent and brief vertigo with corresponding nystagmus when extending or turning the neck, getting up or lying down, or rolling over in bed. In most cases, BPPV is idiopathic, but also can be secondary (following head trauma, viral infection, Meniere’s disease, migraine, otologic and non-otologic surgery, prolonged bed rest [4,5].

It was reported that using canalith repositioning therapy (CRT), such as Epley’s maneuver, otoliths are moved from the semicircular canal to the vestibule where they are absorbed. Complete recovery with resolution of symptoms and disappearance of positional nystagmus after a single maneuver is achieved in about 50–60% of the patients, and after repetitive maneuvers in more than 90% [6]. Posterior semicircular canal involvement is the most common form in BPPV (90–91%), but lateral and anterior canals can also be involved [7].

The BPPV recurrence has been defined as the reappearance of positional vertigo and nystagmus after at least one month from the execution of an effective CRT [8]. The frequent recurrence of BPPV may cause great inconvenience in the daily life of patients with reoccurring BPPV. Many recent studies found that BPPV can be associated with other comorbidity diseases, including hypertension, diabetes, thyroid disorders, hyperlipidemia, and osteoporosis and may be responsible for increased frequency of recurrence of BPPV following treatment [2]. If such a correlation between common comorbidities and BPPV exists, appropriate treatment of these conditions may be useful in limiting chronicity and reducing the frequency of recurrence.

The purpose of this review is to assess the recurrence rate and the time interval, as well as to identify the etiologic factors that may contribute to the recurrence of BPPV. Thus, our aim is to provide an overview of the literature in an area that is still unclear, direct physician’s attention to what factors should assess when encountering patients with BPPV as well as contribute to the knowledge on the mechanisms of the risk factors implicated.

## 2. Materials and Methods

### 2.1. Literature Search

**Figure 1:**
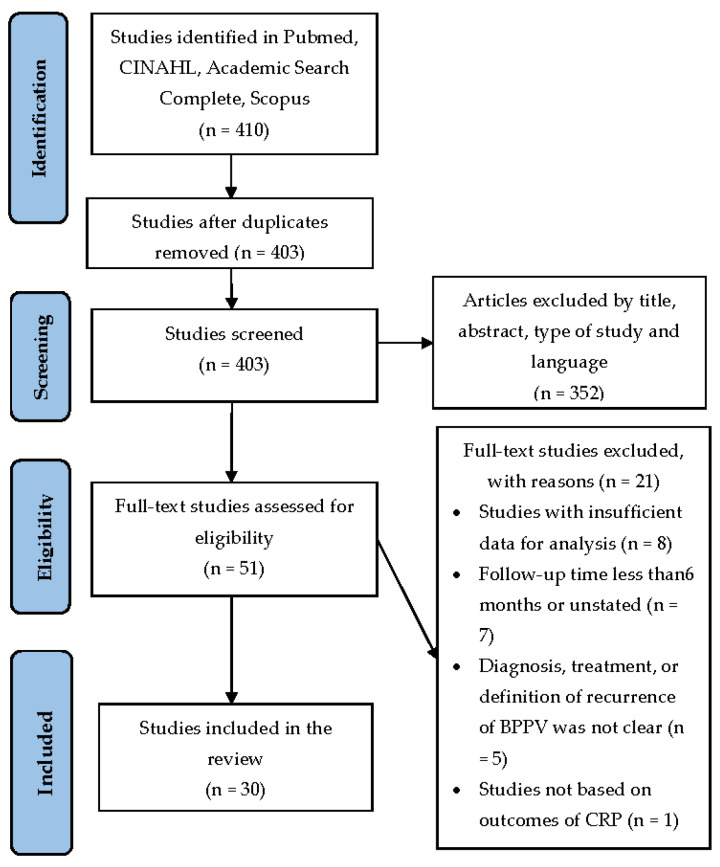
Flowchart of data search and studies selection.

A review of the literature on the risk factors of BPPV recurrence was performed. The articles were identified using MeSH descriptors in the Pubmed database, for studies published until December 2020 (Figure 1); the electronic databases of CINAHL, Academic Search Complete and Scopus were also used for the same period. After combining specific keywords, “BPPV”, “Benign paroxysmal positional vertigo”, “Recurrence”, “Recurrent”, 410 articles were identified. Each title was screened for eligibility according to the predetermined requirements by first author (IS), followed by an independent screening of the abstract and full text by all authors (PB, PK, GGD, GP). All the full texts were verified by the assigned authors. Any discrepancies between the authors’ decisions were addressed through dialogue until a decision was reached.

### 2.2. Eligibility and Exclusion Criteria for Study Selection

The eligibility criteria for inclusion in the analysis were the following:

- Retrospective or prospective studies on the recurrence of BPPV.
- BPPV diagnosis according to clinical practice guideline [9]
- Assessment of the demographics (sex, age) of recurrent BPPV patients and the potential risk factors of BPPV recurrence.
- Articles published in English.

The exclusion criteria in the analysis were the following:

- Systematic reviews, case reports, book chapters.
- Experimental studies.
- Studies not based on outcomes of canalith repositioning procedure (CRP).
- Studies with insufficient data for analysis.
- Diagnosis, treatment, or definition of recurrence of BPPV was not clear.
- Follow-up time less than 6 months or unstated.

### 2.3. Data Extraction

Studies were initially identified on the basis of title and abstract and then assessed for eligibility by full text, in order to establish the final set of studies included. The following data were extracted and evaluated: First author, year of publication, type of study design, population size, mean age, gender, location of BPPV, follow-up time, recurrence rate, time to recurrence, location of recurrence, risk factors assessed.

## 3. Results

### 3.1. Study Selection

A total of 403 articles were initially retrieved from databases (Figure 1). Of these, 352 articles were excluded by title, abstract, type of study and language. Of the remaining 51 articles, after further, full-text evaluation, 21 were excluded according to the abovementioned criteria. Finally, 30 articles met the eligibility criteria for the study (Table 1).

### 3.2. Findings

Thirty articles examined 14 risk factors for recurrence of BPPV and they included 12,585 participants (Table 2). Twenty-three were retrospective studies [1,2,3,4,6,7,8,10,11,12,13,16,18,19,20,21,23,24,25,26,29,30,31] and seven were prospective cohort studies [5,14,15,17,22,27,28]. In all studies, the diagnosis of BPPV was established by positive positional tests (Dix-Hallpike, roll tests). Twenty-nine of the thirty studies provided data on gender of which 7912 of the 12,513 patients were female (62.9%). In one study [14] the proportion of gender was not stated. The mean age of the participants in twenty-seven of the thirty studies was 57.5 years; three studies did not provide sufficient information on the mean age of their participants [14,27,30].

*Initial semicircular canal involvement*. Seven studies [3,6,7,10,17,28,30] examined cases of unilateral posterior canal BPPV; eight studies [8,19,22,23,24,25,26,27] analyzed unilateral BPPV cases involving the three semicircular canals (posterior, horizontal, anterior) and nine studies [1,5,11,15,16,18,20,21,31] reported cases of bilateral or multiple-canal BPPV.

*Follow-up time*. Nine studies [2,13,15,18,20,21,23,24,25] had a follow-up duration greater than 6 months to less than a year, reporting a recurrence rate (RR) in the range of 13.7% [25] to 48% [23]. Furthermore, ten studies [3,4,5,6,7,11,14,27,28,31] had a follow-up time ≥2 years with a recurrence rate varying from 13.3% [11] to 65% [28].

*Time to recurrence*. The majority of the studies -nine out of twelve- found that most of the recurrent episodes developed within the first year [5,6,8,10,15,16,18,19,24]. In particular, Perez et al. [5] reported that 50% of the recurrent episodes occurred within the first 6 months. Luryi et al. [16] and Brandt et al. found [6] that 56% and 80% of recurrences, respectively, developed within the first year and Kim et al. [8] reported that the mean period of recurrence was 11.6 months. On the contrary, Kansu et al. [7] found that most of the patients (53%) experienced recurrence within the first two years whereas two studies [17,28] found that the majority of the recurrent episodes occurred within 4 years after the initial treatment. Specifically, Nunez et al. [17] reported that by 40 months the overall incidence of recurrent episodes approached 50% and Rashad et al. [28] found that the overall mean time to recurrence was 46.3 months.

*Location of recurrence.* Compared to the initial location of BPPV, ipsilateral side was the most affected side in the range of 63% [5] to 76% [16] of the recurrences, while Messina et al. [19] found that 49.2 % of the recurrent episodes developed in the same canal. In addition, Babac et al. [15] reported that 30.65% of the recurrences affected the posterior canal of the contralateral side and Kim et al. [8] found that 16% of the patients suffered recurrence in a different semicircular canal from the one initially involved.

*Analysis of each included risk factor for BPPV recurrence* (Table 2). Patients with hyperlipidemia and hypertension had the highest recurrence rates of BPPV, 67.80% and 55.89%, respectively. Moreover, it seems that more than half of patients (53.48%) with diabetes mellitus and BPPV experienced recurrence of BPPV. In addition, it appears that migraine is also a contributor to the recurrence of BPPV as BPPV recurs in 41.71% of the cases. The rate of BPPV recurrence among the patients who also suffer from osteopenia/osteoporosis and vitamin D deficiency is 40.18% and 38.86%, respectively. Furthermore, our study showed that BPPV recurs in 39.44% of the cases that are comorbid with Meniere’s disease and in 34.81% of the posttraumatic cases of BPPV. Lower recurrence rates have been observed in cases of bilateral/multicanal BPPV (33.33%), in cases of patients with advanced age (32.05%) and in the female patients (32.08%). Sleep disorders and cervical osteoarthrosis had been studied in a small sample size of patients suffering from BPPV recurrence, therefore the conclusions are ambiguous.

## 4. Discussion

### 4.1. Age

Progressive demineralization with advancing age leads to degradation, fragmentation and detachment of utricular otoconia, resulting in BPPV [32]. Additionally, the higher number of recurrences in the elderly patients might be explained by the reduction in daily activities, limited mobility, fatigue and increase in falls [11,15,18]. Whether age represents an independent prognostic factor is controversial. Contrary to studies [7,25] the recurrence rate was demonstrated to be increased with advancing age [4,11]. In studies with large samples, it has been shown that patients older than 40 years [28], or 50 years [15], or 65 years [6] or in the sixth [6] or mainly seventh decade of age [2] were more likely to relapse. Piccioti et al. [4] supported that the risk for recurrence of BPPV was found to be 1.6 times more in patients older than 65 years compared to younger than 65 years; they also noted that the presence of comorbidity (hypertension, diabetes, vascular diseases) might increase the rate of recurrence in aged patients.

### 4.2. Gender

BPPV is frequent in females older than 50 years old [13]. This trend could be attributed to the increased prevalence of osteoporosis and osteopenia in postmenopausal women [33,34], due to the decrease in estrogen secretion (as discussed below). In contrast to age, fewer studies [3,18] have supported that females were more likely to exhibit recurrences of BPPV compared to studies in which the recurrence was shown to not significantly relate to gender [11,28]. However, according to a very recent meta-analysis [33], females were more prone to relapse. Brandt et al. [6] reported higher rate of recurrence in women with a quoted female-to-male ratio 3:2.

### 4.3. Meniere’s Disease

Meniere’s disease is commonly associated with BPPV. However, this association is still ambiguous and could be attributed to the repeated distention of the membranous labyrinth due to hydrops, which may lead to otoconia detachment, loss of resilience and partial collapse of the semicircular canal; the resulted partial obstruction prevents the otoliths from returning to vestibule during the repositioning maneuvers, increasing the rate of treatment failure [35]. Partial obstruction may also be due to a dilated saccule or adhesion of otoliths to the membranous labyrinth [21]. It has been reported that BPPV patients with Meniere’s disease have a 6.009-fold higher risk of recurrence compared to those without Meniere’s disease [18], or a 35% recurrence rate when endolymphatic hydrops and BPPV were associated [10]. However, Luryi et al. [16] did not support the association between BPPV recurrence with pre-existing Meniere’s disease, reporting that signs and symptoms of Meniere’s disease may be conflated with symptoms of concurrent BPPV. Patients with BPPV and Meniere’s disease required more canal repositioning maneuvers than those with idiopathic BPPV without Meniere’s disease [10].

### 4.4. Trauma

Trauma is considered one of the most common causes of secondary BPPV [31,36]. The nature and severity of traumas are diverse and include head trauma, whiplash injury, head and neck surgery. Traumatic BPPV (t-BPPV) has certain distinctive clinical characteristics. In particular, it is associated with a higher incidence of bilateral or multicanal involvement, a greater number of repositioning maneuvers for resolution and a higher recurrence rate [5,12,31,36]. Regarding the persistence of t-BPPV, there is a general consensus that posttraumatic BPPV is more difficult to cure than the idiopathic type (i-BPPV) [4,5,10,12,15,31]. However, there has been a lot of controversy over the recurrence of t-BPPV. Gordon et al. [31] reviewed the clinical records of 21 patients with t-BPPV and compared the outcome with the results of 42 patients with i-BPPV. They found that, during a mean follow-up of 22 months, recurrence was significantly more common in t-BPPV patients (57%) than in idiopathic cases (19%). They also suggested a possible pathogenetic mechanism according to which posttraumatic otoconial detachment and associated microscopic hemorrhage or tissue impairment, resulting in biochemical changes that enhance the reformation of otoconial clots. Kansu et al. [7] in a retrospective study with a long-term follow-up period, found that patients with BPPV caused by head traumas are more likely to relapse compared to i-BPPV patients. In the same line, Choi et al. [12] found that persistent and recurrent BPPV had a higher incidence rate in patients with secondary BPPV including BPPV caused by trauma. Prokopakis et al. [27] in a large prospective study with a mean follow-up of 74 months, also indicated that head trauma, among other causes, increases the risk of recurrence significantly (*p* < 0.001). Perez et al. [5] suggested that cases of traumatic BPPV are considered complex and therefore tend to relapse more often, but found no significant difference in the rate of recurrence. They also suggested that it is the BPPV syndrome that relapses rather than BPPV affecting a particular side or canal. On the other hand, most of the studies in our review did not find a significant correlation between trauma and BPPV recurrence [3,4,6,10,15,16,17].

There are certain difficulties in assessing trauma as a risk factor of recurrence. Firstly, there is high heterogeneity in the type and the severity of trauma, as well as on the intervals between the traumatic event and the diagnosis of BPPV due to other possible medical issues that patients experience after the event. In addition, the repositioning maneuvers in patients with severe traumas could cause pain and discomfort; therefore, in some cases, they are avoided. In most studies of our review, the cohort size was small, and the information provided about the nature of trauma was insufficient. Thus, more prospective studies with more precise data about the history and the clinical characteristics of traumatic BPPV are necessary, in order to clarify the role of trauma in BPPV.

### 4.5. Osteoporosis

Otoconia are a result of inorganic calcium carbonate deposited onto an organic matrix core composed of glycoproteins, mainly otoconin 90 [22,23,37]. Otoconia are in a dynamic state and calcium is required for their mineralization and turnover [38,39]. Calcium and carbonate levels in the endolymph should be kept at a critical level to initiate and maintain the mineralization of the otoconia protein matrix but also avoid unnecessary mineralization [40,41]. This critical balance is achieved by pore-like openings located on the crystalline surface of otoconia, playing an important role in the control of homeostasis [41]. It is speculated that disturbance of calcium metabolism induced by osteoporosis/osteopenia can lead to BPPV by different mechanisms. Vibert et al. [40] found that in ovariectomized osteopenic/osteoporotic female adult rats, otoconia were modified, regarding size and density, suggesting two possible mechanisms. Firstly, they assumed that the reduction in estrogen level can decrease fixation of calcium and subsequently, generate failures in the remodeling of the internal structure of the otoconia as well as in their attachment on the gelatinous matrix. Secondly, an increased concentration of free calcium in the endolymph might induce a reduction in its capacity to dissolve dislodged otoconia and also disturb electromechanical transduction of the sensory epithelium [40,42]. Furthermore, it has been suggested that osteopontin, a bone matrix protein that is considered to form a complex with calcium carbonate crystals at the otolith margin, is reduced in patients with osteoporosis, leading to an impaired otolith formation [43]. Thus, in patients with BPPV and osteoporosis, calcium metabolism failure may be present as a common pathogenesis, leading to the synthesis of atrophic fragile otoliths [13].

There has been controversy about the implication of osteoporosis on the recurrence of BPPV. Babac et al. [15] found that osteoporosis is a potential risk factor for poor treatment results but not for recurrence. Kim et al. found [8] that decreased mineral density (BMD) did not show significant association with BPPV recurrence but showed a significant relation with BPPV occurrence. As a study result similar to Kim et al., Yang et al. [23] found BMD in women is associated with the occurrence of BPPV, though a low BMD and age correlate with the recurrence of BPPV. De Stefano et al. [1], in a multicenter observational study, found that osteoporosis was related to an increased risk of relapse when it was in combination with other comorbidities and defined “groups of risk”. Contrary to the above, a few studies have demonstrated an apparent association between BPPV recurrence and BMD score. Yamanaka et al. [13] did not find a clinical association between BPPV and osteoporosis but the results of his study suggest that osteoporosis is a risk factor for BPPV recurrence and that the prognosis of the BPPV might be clinically predicted by BMD reduction. Talaat et al. [22] in their study found that there was an association between reduced BMD and development/recurrence of BPPV. In a retrospective chart review, Mikulec et al. [44] found a significant negative association between BPPV and treated osteoporosis in women aged 51 to 60 years suggesting the possibility that anti-osteoporotic medication may provide protection against BPPV.

These controversies may be explained by different methodologies to measure osteoporotic changes (location for measuring BMD, bone turnover markers), different definitions of BPPV recurrence and follow- up and various distributions of age or gender in study populations. In particular, most of the studies included women, especially older women because BPPV is more common in women between 41 to 61 years old. In addition, most of the studies are retrospective studies where it is difficult to elucidate causal relationships between the recurrence of BPPV and variables including BMD.

### 4.6. Vitamin D

Vitamin D plays a crucial role in the homeostasis of calcium and phosphorus. Normal serum level of vitamin D is essential for the development of normal otoconia through keeping the calcium concentration in the vestibular endolymph at a normal critical level [45,46]. The epithelial Ca^2+^ channel transport system, Na^+^/Ca^2+^ exchangers, and plasma membrane Ca^2+^ pumps expressed in the inner ear contribute to this critical balance of calcium levels by transepithelial absorption of Ca^2+^ from the endolymph of the inner ear [47]. Vitamin D receptors in the epithelial cells of the inner ear regulate the expression of some Ca^2+^ binding proteins [20,22,23]. Therefore, it has been suggested that vitamin D deficiency also contributes to the development and recurrence of BPPV by abnormal calcium metabolism in the inner ear [23]. Rhim et al. [20] found that vitamin D affects BPPV as a recurrence factor independent of age, gender, follow-up period and type of BPPV. Talaat et al. [22] found that low levels of Vitamin D were related to the development of BPPV while very low levels were associated with recurrence of BPPV. Hence, in the following study, Talaat et al. [48] analyzed the effect of treatment of severe vitamin D deficiency on the recurrence rate of BPPV and found that improvement of serum 25-hydroxyvitamin D3 levels is associated with a substantial decrease in recurrence of BPPV. On the contrary, Yang et al. [23] found that the levels of vitamin D are significantly decreased in men with idiopathic BPPV but they are not associated with the recurrence of BPPV. Similarly, Sreenivas et al. [2] found that the recurrence among patients with vitamin D deficiency was not statistically significant. They assumed that there are significant differences between bone and otoconia formation and this could be partially explained by the fact that the calcium for otoconia formation comes from the endolymph; therefore they speculated that serum markers of turnover are not directly involved in the pathogenesis of BPPV [2].

In most of the studies, blood samples were not obtained at a constant time. Given that there is seasonality of vitamin D, it is critical that future studies should take more parameters into account that affect vitamin D levels such as season, country’s climate, lifestyle and skin color.

### 4.7. Diabetes Mellitus/Hyperinsulinism/Hyperglycemia

Changes in glucose metabolism have been associated with a high prevalence of inner ear disorders, hence with BPPV occurrence and recurrence [14,49,50]. These metabolic disorders can act as a principal etiologic factor in vestibular dysfunction, as well as an aggravating factor of a pre-existing vestibular disorder [51]. Yoda et al. [50] reviewed temporal bones of patients with type 1 diabetes and found that they exhibit a much higher prevalence of migration of otocone debris coming from the utricle, compared to healthy patients. In addition, they found that this difference was associated with the duration of the disease. D’ Silva et al. [52] reported a higher prevalence of BPPV in patients with type 2 diabetes and found that this association was mediated by hypertension. The pathophysiologic mechanism for this correlation is still elusive. Hyperglycemia increases vascular resistance by inhibiting nitric oxide-related vasodilation, thus a combination of hypertension and diabetes may lead to tissue hypoxia and cochleovestibular degeneration [52]. In diabetes the histopathological changes of microangiopathy and vestibular neuropathy are present. Therefore, diabetes-associated neuropathy and vasculopathy as well as microvascular damage, including atherosclerosis, contribute to otoconial degeneration and thus precipitate BPPV [2,19]. In addition, diabetic patients have a poorer capacity for recovery from mild insults, such as viral infections or mild trauma, making these insults more severe [2]. Furthermore, these patients present mutations in the BETA2/NeuroD1 gene which is essential for the normal development of the sensory epithelia of the cochlea, utricle, saccule, and crista ampullaris [53]. On the other hand, hyperinsulinism may disrupt inner ear homeostasis and alter the ionic and metabolic characteristics of the stria vascularis, which is responsible for maintaining endocochlear potential through potassium secretion in the endolymphatic space [49,51]. The inner ear is also affected by hyperinsulinism due to a large number of the insulin receptors present in the endolymphatic sac [14].

In our review, Webster et al. [14] in a prospective study, found that both hyperinsulinism and hyperglycemia behaved as a risk factor for recurrence of idiopathic BPPV and also that a normal glucose tolerance test acted as a protective factor. De Stefano et al. [1] and Messina et al. [19] conducted multicenter observational studies with a large number of subjects. They evaluated the correlation between comorbidities and recurrent episodes of BPPV and found, among other things, that the presence of diabetes is associated with a statistically significant increased risk of recurrence. Piccioti et al. [4] also found that diabetes was associated with the recurrence of BPPV and that in the recurrent group, patients with more than one comorbidity were significantly more numerous compared with patients with one comorbidity. In line with the abovementioned studies, Kim et al. [8] in a smaller study group, found that comorbidities of diabetes and hypertension were associated with recurrence of BPPV. On the other hand, Sreenivas et al. [2] found a significant association between recurrence of BPPV and diabetes mellitus but not hypertension. However, there are few recent studies that found no significant rate of recurrence among diabetic patients. Specifically, Luryi et al. [16] and Zhu et al. [18] in two large single-institution studies of recurrence of BPPV, found no association between recurrence and diabetes mellitus. Wei et al. [24] in a smaller retrospective study, also did not find a correlation between the presence of comorbidities in general and diabetes mellitus in particular and increased recurrence rates of BPPV.

It seems that diabetes mellitus, as with other comorbidities, plays an important role in BPPV occurrence and recurrence. However, most studies were observational studies that did not investigate whether the patients had a good glucose control (hemoglobin A1C levels), or provide information about subcategories of diabetes (Type 1 versus Type 2), resulting in a limitation of potential therapeutic guidance. Therefore, further studies should be conducted in order to evaluate the possibility that proper treatment may reduce the prevalence and recurrence of BPPV.

### 4.8. Vascular Comorbidities (Hypertension, Hyperlipidemia, Cardiovascular Disease)

Numerous epidemiological studies have shown a possible association between BPPV and cardiovascular risk factors [1,4,8,19,26]. These studies support the hypothesis of a vascular role in the aetiopathogenesis of BPPV and its recurrence. Specifically, otoconial detachment from the otolith membrane might be facilitated by microvascular modification and ischemia, further enhanced by hypertensive peaks [4,54]. Furthermore, the blood supply to the inner ear is a terminal circulation, thus any occlusion of the AICA (anterior inferior cerebellar artery) or VBA (vertebrobasilar artery) can cause an ischemic event, leading to audio-vestibular disorders [1,19]. The vestibular system is degraded with age and as a result of changes caused by hypertension and atherosclerosis, resulting in progressive detachment of otoconia from the otolithic membrane [26]. Therefore, multiple systemic diseases increase the recurrence rate [1,4,19].

In this review, most of the studies found that patients with BPPV comorbid with hypertension had an increased recurrence rate of BPPV [1,4,8,18,19,26]. Tan et al. [26] divided the patients into two groups, a group of patients with idiopathic BPPV and hypertension and the second group of patients with idiopathic BPPV. They found a statistically significant difference in the recurrence rate, thus the presence of hypertension is significant for the prognosis of BPPV. De Stefano et al. [1] defined “groups of risk” in order to evaluate whether the combination of multiple conditions further increased the risk of recurrence and found that the combination of hypertension, diabetes, cervical osteoarthrosis and osteoporosis had the highest risk of relapse (HODo risk group). Zhu et al. [18] analyzed the clinical characteristics and risk factors for the recurrence of BPPV in different ages and found that hypertension and hyperlipidemia exhibit a higher risk of recurrence compared to age-matched patients without these diseases. Messina et al. [19] found that among patients with BPPV, 64.5% of patients with hypertension had recurrence; adding that cardiovascular risk factors expose the patients suffering from BPPV to a risk of relapse (OR > 2). Piccioti et al. [4] and Faralli et al. [25] also found that the presence of vascular factors increases the incidence of relapse. On the other hand, Sreenivas et al. [2] found that the presence of hypertension in patients with recurrence of BPPV was not statistically significant (*p* = 0.085). In the same line, Su et al. [3] and Babac et al. [15] did not find an association between recurrence of BPPV and cardiovascular disorders. Similarly, Wei et al. [24] did not detect a correlation between vascular comorbidities and recurrence commenting that this could be due to the short follow-up period of six months.

The role of vascular comorbidities in the recurrence of BPPV is still questionable. There are certain difficulties in quantifying clinical data, especially in observational studies, due to the diversity of comorbidities and the plurality of treatments. Moreover, the follow-up period in most of the studies does not provide the time to detect all events of recurrence. In addition, a lot of patients fail to attend follow-up visits or experience a transient recurrence with a rapid resolution of symptoms. However, it seems that hyperlipidemia and hypertension have a significant association with the recurrence of BPPV. Therefore, appropriate treatment of these conditions may be useful in limiting the frequency of recurrence.

### 4.9. Migraine

Migraine has long been associated with vertigo, with studies suggesting that patients suffering from migraine are two to three times more likely to suffer from vertigo, compared to the headache-free controls [55,56,57]. Moreover, migraine was found to be three times more common in patients with BPPV of an unknown cause than in those with BPPV secondary to trauma or surgical procedures [30]. The mechanisms by which this association arises are not well understood. One of the proposed theories suggests that migraine causes vasospasm of the labyrinthine arteries and the subsequent ischemic damage leads to the release of otoconia from the utricle [30]. Recurrent vasospasm is also associated with the oxidative stress of endothelial cells, which is a possible pathogenetic mechanism common to both migraine and BPPV [58,59]. Therefore, it is speculated that patients with migraines have a recurrent vascular damage in the inner ear that disposes to recurrent BPPV [30,55]. In addition, trigeminal nerve stimulation, an underlying pathophysiologic mechanism of migraine, induces fluid extravasation in the cochlea leading to the detachment of otoconia from the otolith organs [60]. Lastly, it has been reported that certain mutations in a Ca^2+^ channel gene, detected in familial hemiplegic migraine, affect the ion channels of the brain as well as those found in the inner ear, causing imbalance of the resting potentials [30,61].

There are few articles in the literature that address the epidemiological relationship between migraine and the occurrence and recurrence of BPPV, despite the fact that there is a well-recognized association with vertigo. Ishiyama et al. [30] found increased recurrence rates (77%) in the BPPV with migraine group compared to 66% in the group of BPPV without migraine. Zhu et al. [18] in a large study analysis, also reported that BPPV recurrence was associated with migraine (*p* = 0.005). However, Hilton et al. [29], in a large BPPV cohort, compared the recurrence rates between the BPPV with migraine group and the BPPV without migraine group and found no significant difference (38.3% versus 32.1%). In relatively smaller studies, Babac et al. [15] Kansu et al. [7] and Brandt et al. [6] did not find a significantly higher recurrence of BPPV in patients with BPPV and migraine. It is worthwhile to mention the age distribution of patients with BPPV and migraine. In particular, Ishiyama et al. [30] showed that the age of onset in patients with BPPV without migraine was recorded mostly in older age groups, with a peak in the eighth decade. On the contrary, nearly half (47%) of the patients with onset of BPPV before the age of 50 years old had migraines. Similarly, Hilton et al. [29] found that patients over 60 years old had a lower likelihood of having concurrent BPPV and migraine, whereas younger age was independently associated with concurrent comorbidity of BPPV and migraine.

Τhere is a complex relationship between BPPV and migraine due to the high prevalence of vertigo in migraine patients. Vestibular migraine has been recognized as one of the most common causes of recurrent vertigo occurring in migraine patients [62]. In several cases, vestibular migraine may be episodic and appears to be a BPPV reoccurrence, especially when the patients present with central positional nystagmus. Positional nystagmus is of central type and not due to BPPV when a) it has no latency and beats in a direction not aligned for the plane of a particular semicircular canal (e.g., no difference in the nystagmus vector with right versus left Dix-Hallpike), and b) there was no crescendo-decrescendo pattern to the nystagmus and lack of fatigability [62,63]. Distinguishing between the migraines and BPPV is crucial, because multiple maneuvers for misdiagnosed BPPV in a patient with migraines are uncomfortable, distressing and unnecessary if nystagmus is resulting from a migraine. In this case, the treatment should be targeted in the management of migraine episodes [62].

### 4.10. Bilateral/Multicanal BPPV

Bilateral and multiple-canal BPPV are less frequent forms of BPPV that typically require a larger number of maneuvers. Korres et al. [11] found increased recurrence rates in BPPV patients with bilateral canal involvement. Perez et al. [5] defined complex BPPV in cases of either BPPV affecting more than one canal or requiring a large number of maneuvers and found that these patients were at higher risk of recurrence. Moreover, they suggested that the labyrinths of patients with complex BPPV underwent inflammatory changes leading to recurrent episodes of BPPV [5]. Babac et al. [15], in a small group of patients with bilateral and multicanal BPPV, did not detect a negative impact of these factors on BPPV recurrence. Similarly, according to other studies [7,20], a significant correlation between bilateral/multicanal involvement of BPPV and BPPV recurrence was not found. Noticeably, the small number of patients suffering from bilateral/multicanal BPPV is a limitation of reaching reliable conclusions on the impact of these variables on BPPV recurrence, thus further investigation is required.

### 4.11. Cervical Osteoarthritis (Spondylosis)

Cervical spondylosis, also defined as osteoarthritis of the cervical spine, is a common age-related condition. More than 85% of people over the age of 60 are affected by cervical spondylosis [64]. It arises as a result of age-related dehydration of the nucleus pulposus and its collapse, causing bulging of the annulus fibrosus. As the disks dehydrate and shrink, signs of osteoarthritis develop, including bony projections along the edges of bones called osteophytes. These osteophytes cause cord space narrowing [64,65]. As it is reported, the vertebrobasilar circulation in patients with cervical spondylosis is insufficient [65,66]. Therefore, considering the fact that the blood supply to vestibulocochlear organ is an end artery, it is reasonable to assume that reduced blood flow to the labyrinth contributes to the dislodgement of otoconia from the macula of otolith organs [33]. In the literature, there is a deficit of studies examining the effects of cervical spondylosis on BPPV. De Stefano et al. [1] evaluated the relationship between recurrent episodes of BPPV and the most common comorbidities in the elderly population and found that the risk of relapsing BPPV in patients with cervical osteoarthritis increases 3 times; this association became statistically significant when related to the number of recurrences. On the contrary, Wei et al. [24] in a smaller study group with a relatively shorter follow-up period of 6 months, did not detect a correlation between cervical osteoarthritis and increased recurrence rates of BPPV. Li et al. [33] in a systematic review of the risk factor-associated recurrence of BPPV, found that cervical spondylosis, among other systemic diseases, could increase the recurrence of BPPV (*p* < 0.05).

It is possible that cervical spondylosis may favor the recurrence of BPPV. This could be explained by the fact that canalith repositioning procedure (CRP) on patients with dysfunction of the cervical spine is very difficult to be performed, thus leading tο improper treatment or early recurrence of BPPV. Martelucci et al. [67] investigated the impact of reduced cervical mobility on CRP efficacy and suggested three pathophysiological mechanisms: (1) Debris could remain in the canal lumen and then return to the ampullary arm, causing the failure of the maneuver. (2) Part of debris could leave the canal while the rest could sprinkle in the endolymph and then accumulate again. Therefore, a transient regression of symptoms followed by an early relapse can occur. (3) The otoconial could relocate into the superior or horizontal semicircular canals, leading to a canal switch.

### 4.12. Sleep Disorders

Sleep disorders, especially insomnia, are associated with numerous physical and psychiatric health problems [68]. The pathophysiological link between BPPV and sleep disorders is still unclear. It is suggested that bad sleep leads the patients to multiple head movements during the night, therefore dispose them to a higher risk of BPPV relapse [3]. Other potential mechanisms include neuroendocrine dysfunction, caused by increased cortisol levels, as well as activation of inflammation of the nervous system, including vestibular neurons [69]. In addition, about 40% of individuals with insomnia have a comorbid psychiatric condition like anxiety and depression [69], which is known that may serve as the primary cause of vestibular symptoms as well as a risk factor for BPPV recurrence [70,71].

Only one study [3] analyzed the relationship between insomnia and BPPV recurrence; in this study, Su et al. [3] found that 30.4% of the participants in the recurrence group reported sleep disorders, the majority of which suffered from chronic insomnia and were under medication. Respectively, in the non-recurrence group, 13.2 % exhibited sleep disorders. The difference between the two groups was found to be significant.

## 5. Limitations

There are several limitations in our review. First of all, there was a high degree of heterogeneity between the studies. In particular, there were differences in the design of the studies such as duration of follow-up and cohort size. In addition, there was a diversity of the risk factors concerning diagnostic criteria of the comorbidities, quantification of their clinical data, as well as evaluation on disease control. Additionally, there was a small number of studies for some risk factors (bilateral/multicanal BPPV, cervical spondylosis, sleep disorders), which might have limited the reliability and validity of our results. Another important limitation was that most of the studies were retrospective studies, thus it was more difficult to elucidate causal relationships between the recurrence of BPPV and the risk factors. Last but not least, our review was a descriptive review and we did not assess the quality of the articles, nor did we use systematic review tools. Our intention was to avoid complex methods that in many cases are difficult to be interpreted by physicians and provide a clinical perspective on the recurrence of BPPV.

## 6. Conclusions

This comprehensive review evaluated some possible risk factors for BPPV recurrence as there is no general consensus in the literature concerning their significance. Our principal aim was to direct physician’s attention to what factors should be assessed when encountering patients with BPPV. Therefore, a clinical practitioner should, first of all, take a good medical history. Patients with cardiovascular comorbidities, especially hyperlipidemia and hypertension, as well as patients with diabetes mellitus have an increased risk of BPPV recurrence. Thus, good control of these comorbidities should always be under consideration in these patients. In addition, osteoporosis and vitamin D deficiency should be probed when treating postmenopausal women with recurrent episodes of BPPV. Additionally, a typical blood test could be useful in order to provide information on a patient’s lipidemic and glycemic profile as well as to reveal a possible vitamin D deficiency. Migraine and Meniere’s disease have a complex relationship with BPPV; therefore, it is crucial for a physician to distinguish BPPV from these conditions in order to avoid unnecessary and distressing maneuvers. Trauma, advanced age and female gender do not seem to play an important role in BPPV recurrence, unless they are combined with other comorbidities, as mentioned above. Bilateral/multicanal BPPV, cervical spondylosis and sleep disorders are interesting, underestimated risk factors that need further investigation. In conclusion, identification of these risk factors contributes to the evaluation of BPPV patients, in order to establish a long-term prognosis, and helps physicians counseling patients regarding their expectations for the follow-up period.

In the future, more large-scale prospective studies are needed in order to clarify the role of these factors as well as pave the way for new therapeutic strategies.

## Figures and Tables

**Table 1:** Assessed characteristics of the included studies.

Authors	Year	Study	Patients (M/F)	Mean Age	Location of BPPV	Follow up	Recurrence Rate	Time to Recurrence	Location of Recurrence	Risk Factors Assessed
Del Rio et al. [10]	2004	R	104 (35/69)	64.3 y	PSC	6–15 mo	22.6% (21/93)	118 d	N/A	RF3 *:7/16 ** RF4:1/12
Korres et al. [11]	2006	R	143 (62/81)	59.9 y	3SC bilateral multicanal	24 mo	13.3 % (19/143)	N/A	PSC HSC bilateral 2-canals	RF1 *:19/124 RF2: 11/81 RF4: 1/4 RF12 *:5/8
Kansu et al. [7]	2010	R	118 (44/74)	51.8 y	PSC	53–78 mo	33.1% (39/118)	53.8% within 2 y 33.1% within 5 y	PSC bilateral	RF1: 39/79 RF2: 25/74 RF3 *: 3/8 RF4 *:12/25 RF11:5/16 RF12: 2/5
Choi et al. [12]	2012	R	120 (43/77)	50.4 y	PSC HSC	6–29 mo	10% (12/120)	N/A	PSC HSC	RF3 *: 2/2 RF4*: 2/8
Yamanaka et al. [13]	2013	R	61 (0/61)	63.7 y	N/A	12 mo	56% (9/16)	N/A	N/A	RF5 *: 9/16
Webster et al. [14]	2014	P	72 (N/A)	N/A	N/A	41 mo	36.1% (26/72)	N/A	N/A	RF7 *
De Stefano et al. [1]	2013	R	1092 (407/685)	72.9 y	3SC multicanal	6–24 mo	50.5% (551/1092)	N/A	PSC (36.8%)	RF5: 1/ 13 RF7 *: 10/16 RF8 *: 100/163 RF13 *: 8/13
Babac et al. [15]	2014	P	400 (119/281)	58.7 y	3SC bilateral multicanal	12 mo	15.5% (62/400)	48.4% within 3 mo	ipsilateral PSC (61.29%) contralateral PSC (30.65%) multicanal ipsilateral (1 case)	RF1(>50 y): 51/254 RF2: 48/281 RF4: 4/18 RF5: 12/65 RF11: 6/55 RF10: 36/224 RF12: 0/5
Su et al. [3]	2016	R	243 (47/19)	57.5 y	PSC	24 mo	18.9% (46/243)			RF1(>65 y): 15/58 RF2 *: 42/196 RF4: 2/11 RF10: 20/80 RF14 *: 14/40
Kim et al. [8]	2017	R	198 (59/139)	62.9 y	3SC	≥12 mo	33.8% (67/198)	mean period of 11.6 mo	16% in different SC	RF1: 67/131 RF2: 49/139 RF5: 7/14 RF7 *:14/17 RF8 *:27/32
Luryi et al. [16]	2018	R	1105 (315/790)	64.8 y	3SC bilateral	602 d	37%	76% within 2 y	28% in the same ear	RF1(>60 y):267/700 RF2 *: 319/790 RF3: 31/103 RF4: 27/82 RF7: 47/134
Nunez et al. [17]	2000	P	151 (44/107)	63.0 y	PSC	15.9 mo	26.8% (37/138)	15% per year 40% within 40 mo	PSC	RF4: 10/27
Brandt et al. [6]	2009	R	125 (49/76)	55.1 y	PSC	10.3 y	50%	80% within 1 y 94% within 5 y		RF1: 37/54* RF2 *: 44/76 RF4: 7/17 RF11: 8/15
Perez et al. [5]	2012	P	69 (25/44)	62.0 y	3SC bilateral multicanal	47 mo	27% (19/69)	50% within 6 mo	7/19 contralateral ear 5/7 other canal 6/12 other canal	RF1: 19/69 RF2 RF4 *: 3/6 RF12 *: 8/17
Zhu et al. [18]	2019	R	1012 (316/696)	54.6 y	PSC HSC multicanal	12 mo	33,7% (255/757)	12 mo		RF1(>60 y): 91/315 RF2 *: 187/696 RF3 *: 16/24 RF7: 24/87 RF8 *: 89/276 RF9 *: 84/266 RF10: 77/258 RF11 *: 14/29
Messina et al. [19]	2017	R	2682 (1008/1571)	59.3 y	3SC	≥6 mo	52.5% (1386/2638)	1–5 episodes per year (84.3%)	49.2% same canal	RF7 *: 261/413 RF8 *: 844/1401 RF9 *: 1401/1906 RF10 *: 82/265
Sreenivas et al. [2]	2019	R	71 (31/40)	49.0 y	N/A	6–12 mo	22.5% (16/71)			RF1(>60 y) *: 5/11 RF6: 12/56 RF7 *: 5/8 RF8: 8/21 RF9: 10/33
Rhim et al. [20]	2016	R	232 (63/169)	50.35 y	3SC bilateral multicanal	10.2 mo	17.7% (41/232)	N/A	N/A	RF1:41/191 RF2: 31/169 RF6 *:41/191 RF12: 6/28
Balatsouras [21]	2012	R	262 (97/165) 233 iBPPV 29 mBPPV	53.4 y	3SC bilateral multicanal	12 mo	44.4% (iBPPV) 13.3% (mBPPV)	N/A	N/A	RF3 *: 12/27
Talaat et al. [22]	2014	P	80 (28/52)	47.6 y	3SC	≥12 mo	45% (36/80)	N/A	N/A	RF1: 36/44 RF2: 27/52 RF5 *: 22/48 RF6 *: 34/72
Yang CJ et al. [23]	2017	R	130 (30/100)	54.9 y	3SC	12 mo	48% (63/130)	N/A	N/A	RF1 *: 63/67 RF2: 50/100 RF5: 39/68 RF6: 63/67
Wei et al. [24]	2018	R	127 (46/81)	53.9 y	3SC	6 mo	14.17%	6 mo	N/A	RF1(>60y) RF2 RF7 RF8 RF9 RF11 RF13
Faralli et al. [25]	2006	R	566 (204/362)	56.8 y	3SC	6 mo	13.7% (77/560)	N/A	N/A	RF1(>50 y): 64/396 RF10 *: 22/111
Piccioti et al. [4]	2016	R	475 (188/287)	61.9 y	PSC HSC	30 mo	23.4% (139/475)	N/A	N/A	RF1 *: 139/475 RF2 *: 95/287 RF4: 13/42 RF7 * RF8 * RF9 * RF10 *
Tan et al. [26]	2016	R	88 (33/55) 47 i-BPPV 41 h-BPPV	53.44 y (h-BPPV) 51.64 y (i-BPPV)	3SC	≥12 mo	12.7% (6/47, i-BPPV) 31.7% (13/41, h-BPPV)	N/A	N/A	RF8 *: 13/41
Prokopakis et al. [27]	2012	P	965 (481/484)	N/A	3SC	74 mo	15.5% (139/895)	N/A	N/A	RF1(>70 y) * RF4*
Rashad et al. [28]	2009	P	103 (45/58)	48.2 y	PSC	5 y	65% (67/103)	46.3 mo	N/A	RF1(≥40 y) *: 49/82 RF2: 34/58
Hilton et al. [29]	2020	R	1481 (389/1092)	63.3 y	N/A	≥6 mo	33.8% (267/791)	N/A	N/A	RF11: 80/209
Ishiyama et al. [30]	2000	R	247 (93/154)	N/A	PSC	≥6 mo	68.4% (169/247)	N/A	N/A	RF11 *: 48/62
Gordon et al. [31]	2004	R	21 t- BPPV (10/11) 42 i-BPPV (10/32)	56.3 y (t-BPPV) 61.1 y (i-BPPV)	PSC HSC bilateral	6–42 mo	57% (12/21, t-BPPV) 19% (8/42, i-BPPV)	N/A	N/A	RF4 *: 12/21

* Statistically significant Risk Factors. RF1: Advanced age, RF2: Female gender, RF3: Meniere’s disease/Hydrops, RF4: Head trauma, RF5: Osteopenia/Osteoporosis, RF6: Vitamin D deficiency RF7: Diabetes mellitus/Hyperinsulinism/Hyperglycemia, RF8: Hypertension, RF9: Hyperlipidemia, RF10: Cardiovascular disease, RF11: Migraine, RF12: Bilateral/ Multicanal BPPV, RF13: Cervical osteoarthrosis, RF14: Sleep disorders. N/A: Not available, R: Retrospective study, P: Prospective study, PSC, HSC: posterior, horizontal semicircular canal, SC: semicircular canals, y: years, mo: months, d: days, t-BPPV, i-BPPV: traumatic, idiopathic benign paroxysmal positional vertigo. ** In the last column, the number of patients with reoccurring BPPV is shown among the total number of patients having the corresponding risk factor.

**Table 2:** Analysis of each included risk factor for BPPV recurrence in this review.

	N of Studies Assessed	N of Studies with Statistical Significance	Recurrence/ Non-Recurrence (Cases)	Recurrence (%)
Advanced Age	18	6	976/2069	32.05%
Female gender	15	5	962/2037	32.08%
Meniere’s disease	6	5	71/109	39.44%
Trauma	12	4	94/176	34.81%
Osteopenia/Osteoporosis	6	2	90/134	40.18%
Vitamin D deficiency	4	2	150/236	38.86%
Diabetesmellitus/Hyperisulinism/Hypergl-ycemia	9	6	361/314	53.48%
Hypertension	8	6	1081/853	55.89%
Hyperlipidemia	5	3	1495/710	67.80%
Cardiovascular disease	6	3	237/701	25.27%
Migraine	7	2	161/225	41.71%
Bilateral/multicanal BPPV	5	2	21/42	33.33%
Cervical osteoarthrosis	2	1	8/5	61.54%
Sleep disorders	1	1	14/26	35.00%

N: Number.

## Data Availability

The data presented in this study are available upon request from the corresponding author.
